# Genetic ablation of smooth muscle K_IR_2.1 is inconsequential to the function of mouse cerebral arteries

**DOI:** 10.1177/0271678X221093432

**Published:** 2022-04-11

**Authors:** Paulina M Kowalewska, Jacob Fletcher, William F Jackson, Suzanne E Brett, Michelle SM Kim, Galina Yu Mironova, Nadia Haghbin, David M Richter, Nathan R Tykocki, Mark T Nelson, Donald G Welsh

**Affiliations:** 1 Robarts Research Institute and the Department of Physiology & Pharmacology, University of Western Ontario, London, ON, Canada; 2 Department of Pharmacology and Toxicology, Michigan State University, East Lansing, MI, USA; 3 Department of Pharmacology, University of Vermont, Burlington, VT, USA

**Keywords:** Arterial spin-labeling MRI, cerebral blood flow, electrophysiology, myography, potassium channels

## Abstract

Cerebral blood flow is a finely tuned process dependent on coordinated changes in arterial tone. These changes are strongly tied to smooth muscle membrane potential and inwardly rectifying K^+^ (K_IR_) channels are thought to be a key determinant. To elucidate the role of K_IR_2.1 in cerebral arterial tone development, this study examined the electrical and functional properties of cells, vessels and living tissue from tamoxifen-induced smooth muscle cell (SMC)-specific K_IR_2.1 knockout mice. Patch-clamp electrophysiology revealed a robust Ba^2+^-sensitive inwardly rectifying K^+^ current in cerebral arterial myocytes irrespective of K_IR_2.1 knockout. Immunolabeling clarified that K_IR_2.1 expression was low in SMCs while K_IR_2.2 labeling was remarkably abundant at the membrane. In alignment with these observations, pressure myography revealed that the myogenic response and K^+^-induced dilation were intact in cerebral arteries post knockout. At the whole organ level, this translated to a maintenance of brain perfusion in SMC *K_IR_2.1^−/−^* mice, as assessed with arterial spin-labeling MRI. We confirmed these findings in superior epigastric arteries and implicated K_IR_2.2 as more functionally relevant in SMCs. Together, these results suggest that subunits other than K_IR_2.1 play a significant role in setting native current in SMCs and driving arterial tone.

## Introduction

Cerebral blood flow (CBF) is maintained by a network of resistance arteries that match red blood cell delivery with metabolic activity.^1,2^ The vascular smooth muscle (VSM) layer translates vasoactive stimuli into diameter changes, allowing for precise tuning of blood flow needed for optimal brain function.^3–5^ Smooth muscle cell (SMC) contractility is tightly coupled to intracellular [Ca^2+^], an essential secondary messenger that sets myosin light chain phosphorylation and consequently the extent of cross-bridge cycling.^6^ The influx of extracellular Ca^2+^ is facilitated by voltage-gated Ca^2+^ channels (VGCCs), integral membrane proteins whose activity is coupled to arterial membrane potential (V_M_).^7^ It is the balance of inward depolarizing currents and outward hyperpolarizing currents that determine V_M_ in smooth muscle. The latter is largely delivered through membrane-embedded K^+^ channels, including voltage-gated (K_V_), ATP-sensitive (K_ATP_), Ca^2+^-activated, and inwardly rectifying (K_IR_) channels.^8^

In cerebral arteries, K_IR_ channels are major contributors to myogenic tone development.^9^ They exist as tetrameric assemblies of 4 α-subunits from the strong inwardly rectifying K_IR_2.x subfamily, of which K_IR_2.1 is thought to be the predominant subunit in SMCs.^10,11^ K_IR_ channels possess several unique electrical characteristics, including inward rectification at membrane potentials negative to E_K_, potentiation by extracellular K^+^, and rapid blockade by micromolar Ba^2+^.^12,13^ At a functional level, these properties enable K_IR_ channels to participate in K^+^-induced dilation, vessel hyperpolarization, and hemodynamic force sensing.^3,14,15^ Interestingly, K_IR_2.2 subunits have also been identified in cerebral vessels and are thought to form functional channels when heteromultimerized with K_IR_2.1.^15,16^ This knowledge raised a key question centered on the overall contribution of K_IR_2.1 to native currents and their control over cerebral arterial function. Early studies using a global knockout mouse^10,17^ implied a dominant role for SMC K_IR_2.1 in setting arterial V_M_ and tone. While informative, it is noteworthy that experimentation was limited to neonates due to the lethality of global gene deletion. More recent advances in genomic techniques allow for generation of inducible tissue-specific knockouts; this, in turn, creates the ideal opportunity to pursue a deeper understanding of K_IR_2.1 in adult cerebral vasculature.

Building on prior work, this study used an adult tamoxifen-induced SMC-specific K_IR_2.1 knockout model to further explore this channel’s role in cerebral arterial function. Experiments progressed from isolated cells to live animals, employing electrophysiology, immunofluorescence labeling, western blotting, vessel myography, and arterial spin-labeling magnetic resonance imaging (ASL-MRI). Contrary to expectations, we observed robust K_IR_ currents in SMCs isolated from control and knockout mice. Immunolabeling subsequently revealed that, while K_IR_2.1 expression was reduced by knockout, its apparent abundance was low in control animals in contrast to the K_IR_2.2 subunit. Myography next showed that the myogenic response and K^+^-induced dilation remained intact in knockout mice. This K^+^-induced dilation was sensitive to low Ba^2+^ concentration, a property consistent with the electrophysiological profile of K_IR_2.2 rather than K_IR_2.1.^16^ Without a significant change in tone with SMC K_IR_2.1 deletion, brain perfusion was unaffected in live mice, as observed by ASL-MRI. The electrophysiology, protein expression, and myography findings were further confirmed in a second vascular network—epigastric arteries. Taken together, these results indicate that inward K^+^ currents in cerebral arterial myocytes are likely derived from subunits other than K_IR_2.1.

## Methods

### Animal model and ethical approval

All animal procedures followed regulations set by the Canadian Council of Animal Care and were approved by the University of Western Ontario Animal Care Committee (Protocol #2017-144). Colonies were maintained under constant room temperature and humidity environment with a 12-hr light/dark cycle and allowed ad libitum access to food and water. The colony of SMMHC-CreER^T2^ male mice, strain B6.FVB-Tg(Myh11-cre/ERT2)1Soff/J, was derived from a colony at the University of Vermont (Burlington, VT, USA). The SMMHC-CreER^T2^ mice, which express a Cre recombinase under control of the smooth muscle myosin, heavy polypeptide 11, smooth muscle (*Myh11*) promoter on the Y chromosome,^18^ were crossed with floxed K_IR_2.1 mice (strain B6.Cg-Kcnj2^tm1swz^)^19^ to produce tamoxifen-inducible SMC *K_IR_2.1^−/−^* male mice. Six-week-old mice were treated with tamoxifen by oral gavage (0.12 mg kg^−1^ in corn oil; Sigma-Aldrich) once daily for 5 days to induce SMC-specific deletion of K_IR_2.1 via tamoxifen-mediated nuclear translocation of Cre recombinase (Figure S1). Non-induced Cre SMC mice (Cre carriers with the floxed K_IR_2.1 gene) served as control animals. ARRIVE guidelines for reporting animal research were followed.^20^

### Cerebral artery and smooth muscle cell isolation

SMC *K_IR_2.1^−/−^* mice were euthanized via CO_2_ asphyxiation 2–3 months after tamoxifen treatment with age-matched controls. The brain was removed and placed in chilled phosphate-buffered solution (PBS; pH 7.4) containing (in mM): 137 NaCl, 2.7 KCl, 10 Na_2_HPO_4_, 1.8 KH_2_PO_4_, and 5 glucose. Cerebral and cerebellar arteries were carefully dissected and cleaned for single-cell and isolated-vessel experiments. Specifically, middle and posterior cerebral as well as cerebellar arteries were used for patch experiments and posterior cerebral and cerebellar arteries were used for myography work. Dissected arteries were enzymatically digested to yield isolated SMCs using a two-step process.^21^ Briefly, vessel segments were placed in isolation medium containing (in mM): 60 NaCl, 80 sodium glutamate, 5 KCl, 2 MgCl_2_, 10 glucose, and 10 HEPES with 1 mg mL^−1^ bovine serum albumin (pH 7.4), and chilled on ice for 10 minutes before the reaction began. Following an initial warming period (37 °C, 10 minutes), vessels underwent a two-step digestion process: 1) a 6-minute incubation in isolation medium containing 0.9 mg mL^−1^ papain and 1 mg mL^−1^ dithiothreitol and 2) a 7-minute incubation in isolation medium containing 0.3 mg mL^−1^ H-type collagenase, and 0.7 mg mL^−1^ F-type collagenase. Vessels were then thoroughly washed with ice-cold isolation medium and allowed to rest on ice for 30 minutes before disruption with a fire-polished pipette.

### Electrophysiology

Whole-cell patch clamp electrophysiology was used to measure Ba^2+^-sensitive K_IR_ currents in isolated cerebral artery SMCs. Recording electrodes were pulled from borosilicate glass (Sutter Instruments) using a micropipette puller (Narishige PP-830), fire-polished (Narishige MF-830) and filled with pipette solution containing (in mM): 5 NaCl, 35 KCl, 100 K-gluconate, 1 CaCl_2_, 0.5 MgCl_2_, 10 HEPES, 10 EGTA, 2.5 Na_2_-ATP, and 0.2 GTP (pH 7.2). Whole-cell access was obtained by placing the pipette onto a cell and applying negative pressure until membrane rupture occurred. Cells were then voltage clamped at −50 mV and equilibrated in a bath solution containing (in mM): 140 NaCl, 5 KCl, 0.5 MgCl_2_, 10 HEPES, 10 glucose, and 0.1 CaCl_2_. To stimulate K_IR_ activity, extracellular K^+^ was increased with bath solution containing (in mM): 60 KCl, 85 NaCl, 0.5 MgCl_2_, 10 HEPES, 10 glucose, and 0.1 CaCl_2_ (pH 7.4). Voltage was then stepped to −100 mV for 100 ms and subsequently ramped to +20 mV at a rate of 0.04 mV ms^−1^. Ba^2+^ (100 µM), a selective inhibitor of K_IR_ channels, was then added to the bath to distinguish K_IR_ activity from whole-cell currents. Currents were recorded on an Axopatch 200B amplifier (Molecular Devices), with signals filtered at 1 kHz and digitized at 5 kHz. Data were extracted and analysed using Clampfit 10.3 software (Molecular Devices). Cell capacitance ranged from 10 to 16.5 pF as measured by the amplifier. A 1M NaCl-agar salt bridge was added between the reference electrode and bath solution to minimize offset potentials. Experiments were performed at room temperature (21 °C).

### Immunofluorescence

Isolated cerebral arterial SMCs from non-induced Cre SMC control and SMC *K_IR_2.1^−/−^* mice were probed for K_IR_2.1 and K_IR_2.2 protein expression. Cells were settled onto poly-L-lysine-treated cover glass and fixed in 4% paraformaldehyde (15 min, 21 °C). Fixed cells were then washed 3 times with PBS prior to permeabilization with 0.2% Tween 20 (15 min, 21 °C). A quench solution containing 0.2% Tween 20 and 3% donkey serum was used to block cells for one hour (21 °C). Rabbit polyclonal primary antibodies against K_IR_2.1 (APC-026, 1:200; Alomone) and K_IR_2.2 (APC-042, 1:200; Alomone) were diluted in quench solution and applied to cells for overnight incubation (4 °C). The next day, cells were washed with 0.2% Tween 20 and treated with Alexa Fluor® 488 donkey anti-rabbit IgG (A-21206, 1:1000; ThermoFisher Scientific) fluorophore-conjugated secondary antibodies (1 hour, 21 °C). After additional washes, cells were mounted on slides with ProLong™ Diamond Antifade Mountant (ThermoFisher Scientific) with DAPI. Images were captured using a Leica-TCS SP8 confocal microscope (Wetzlar) with a 63× oil-immersion lens. Laser intensity was increased to visualize K_IR_2.1 staining as it exhibited a weak fluorescent signal. Controls for each stain were prepared by omitting the primary antibody. Mean fluorescence of each cell was measured using Image-J and background signal was subtracted using secondary antibody controls. We previously validated the specificity of anti-K_IR_2.1 and anti-K_IR_2.2 by pre-incubation with their corresponding blocking peptide antigen before addition to cells.^15^ SMCs from mesenteric arteries were also used as K_IR_2.x negative controls for these antibodies.^15^

### Western blot analysis

Isolated cerebral arteries were manually ground in ice-cold lysis buffer (150 μL; Tissue Protein Extraction Reagent, ThermoFisher Scientific) with protease inhibitors (Protease Inhibitor Cocktail, Sigma-Aldrich). The supernatant was assayed for total protein using Pierce BCA Protein Assay (ThermoFisher Scientific). The samples were boiled for 10 min in Laemmli buffer containing 65 mM Tris HCL, 10% glycerol, 3% SDS, 0.6 mM Bromophenol blue and 2.5% 2-mercaptoethanol. Approximately 1 μg of protein was loaded into an SDS-PAGE gel with a 4% to 10% gradient and run for 1.5 hrs at 100 V at 21 °C (Bio-Rad). The gel was soaked in transfer buffer, set on a nitrocellulose membrane and left running at 25 V overnight at 4 °C. The membrane was blocked with 5% non-fat dairy milk in Tris-buffered 0.1% Tween 20 (TBS-T) saline for 1 hr. After washing in 0.1% TBS-T, the membrane was incubated with rabbit polyclonal anti-K_IR_2.2 (APC-042, 1:400; Alomone) in 1% milk TBS-T for 2 hrs at 21 °C. After a TBS-T wash, the membrane was incubated with goat polyclonal anti-rabbit horseradish peroxidase-conjugated secondary antibodies (65-6120, 1:10000; ThermoFisher Scientific) in 1% milk TBS-T for 1 hr at 21 °C. For standard controls, membranes were incubated with anti-pan actin rabbit polyclonal antibody (AAN01, 1:1000; Cytoskeleton Inc.) in 1% milk TBS-T for 2 hrs at 21 °C. After washing, the blot was developed using Amersham ECL Prime Western Blotting Reagent (Cytiva, Global Life Sciences Solutions) and imaged on a Bio-Rad ChemiDoc™ MP Imaging System. Densitometric analysis was performed using Image Lab 6.1 (Bio-Rad). Protein signal was normalized to actin levels.

### Cerebral vessel myography

Cerebral arteries were cannulated in a custom mounting chamber filled with physiological salt solution (PSS) containing (in mM): 119 NaCl, 4.7 KCl, 20 NaHCO_3_, 1.7 KH_2_PO_4_, 1.2 MgSO_4_, 1.6 CaCl_2_, and 10 glucose. Vessels were filled with Ca^2+^-PSS and an air bubble was passed from the cannula to strip the vessel of functional endothelium. Bath solution was continuously bubbled with air and maintained at 36.0 ± 0.2 °C. Following equilibration at 15 mmHg for 30 min, 60 mM K^+^ was superfused to assess vessel viability and measure minimum diameter. Absence of intact endothelium was confirmed by exposure to bradykinin (10 μM; Sigma Aldrich). After washing with Ca^2+^-PSS, the internal pressure of the vessel was adjusted to 20 mmHg and then increased stepwise to 100 mmHg in increments of 20 mmHg with 5 min per step. At 100 mmHg, bath K^+^ was increased from 5 mM to 10 mM for approximately 1 min to assess K^+^-induced dilation. After excess K^+^ was washed off, pressure was reduced to 20 mmHg and 100 µM Ba^2+^ was added to the superfusate. The pressure challenge was repeated and, after recording the response at 100 mmHg, vessels were reassessed for K^+^-induced dilation. In a separate group of control animals, dilation to 10 mM K^+^ was assessed before and after addition of 1 μM, 3 μM and 10 μM Ba^2+^. Passive vessel diameter was measured while repeating pressure steps in the presence of 2 mM EGTA in Ca^2+^-free PSS. The diameter was measured using an automated edge detection system (IonOptix) under a 10× objective on a Zeiss Axiovert 200 microscope (Carl Zeiss).

### Arterial spin-labeling magnetic resonance imaging of the brain

Under 2.2% isoflurane anesthesia, a polyethylene catheter (PE10; Instech Laboratories) was implanted intraperitoneally and connected to a syringe containing 1 mM phenylephrine. The animal was placed in a custom-built insert in the prone position and inserted in an Agilent Animal MRI scanner with a 9.4-Tesla, 31-cm horizontal bore magnet (Magnex Scientific), 60-mm gradient coil set of 1000 mT/m strength (Agilent), and Bruker Avance MRI III console with Paravision-6 software (Bruker BioSpin Corp). A 40-mm millipede volume coil (Agilent) was used for data acquisition.

An anatomical reference scan was acquired using a 2D fast spin echo (Turbo-Rapid Acquisition with Relaxation Enhancement (RARE)) sequence with the following parameters: field of view (FOV) = 19.2 × 19.2 mm^2^, matrix size = 128 × 128, 11 slices with slice thickness of 1 mm, repetition time = 5000 ms, echo time (TE) = 10 ms, effective echo time = 40 ms, RARE factor = 8, number of averages = 1.

A flow-sensitive alternating inversion-recovery spin echo planar imaging sequence with a 180° hyperbolic secant radiofrequency inversion pulse was used (imaging parameters: TE = 17 ms; 5 slices with imaging slice thickness of 2 mm; image matrix = 64 × 50; FOV =19.2 × 15 mm^2^, inversion parameters: inversion slab thickness = 13 mm; pulse length = 3 ms) for perfusion images. Eleven images with increasing inversion times (100 ms + i 300 ms (i = 0, 2, 3, …, 10)) were obtained for each slice to determine T_1_. Images with slice selective inversion were acquired followed by images with nonselective inversion. From these images, T_1sel_ and T_1nonsel_ were calculated using a non-linear least square fit (ASL-Perfusion Processing; Bruker). The five 2-mm slices spanned the mouse brain with a total scan time of approximately 13 min.

During acquisition, the animal was maintained at ∼1.75% isoflurane and breath rate was monitored (PC-SAM model #1025; SA Instruments, Inc.) with a pneumatic pillow. Body temperature was measured with a rectal probe and maintained at ∼37 °C with a homeothermic warm air blower. Mean arterial pressure (MAP) was measured with a tail cuff (CODA™ Monitor, Kent Scientific) during MRI acquisition. After a baseline scan of the brain was acquired, phenylephrine hydrochloride (0.816 mg kg^−1^; Sigma-Aldrich) was injected and the scan was repeated. Following completion of MRI, mice were euthanized via cervical dislocation under deep anesthesia. CBF (mL/100 g/min) was quantified in the cortex, cerebral nuclei, hippocampus, thalamus, hypothalamus and midbrain using custom software written in MATLAB (Mathworks, Inc.).

### Animal preparation for superior epigastric artery isolation

All experiments were approved and conducted in accordance with the guidelines set by the Institutional Animal Care and Use Committees at the University of Vermont and Michigan State University. Superior epigastric arteries (SEAs) were isolated from euthanized C57BL/6 (control) and tamoxifen-inducible SMC-specific K_IR_2.1 knockout (SMC *K_IR_2.1^−/−^*) mice as described.^22–24^ For administration of tamoxifen, 6-week-old SMC *K_IR_2.1^−/−^* mice were anesthetized with isoflurane (3% in O_2_), and the interscapular area was shaved and sterilized. A small, subcutaneous pocket was made with blunt dissection and tamoxifen citrate extended-release pellets (5 mg total with 21-day release; Innovative Research of America) were implanted, followed by wound closure. Animals were euthanized a minimum of 4 weeks after pellet implantation for tissue collection.

### Electrophysiology and qRTPCR

SEAs were enzymatically dissociated to yield single SMCs for perforated-patch recording of Ba^2+^-sensitive K^+^ currents to quantify the functional expression of SMC K_IR_ channels, and for qRTPCR as described.^22–24^ For detailed description of electrophysiology, see supplemental material. Briefly, settled SMCs in the recording chamber were superfused with PSS. Pipettes were applied to the cell surface and after electrical access to cytoplasm was attained, cells were superfused with PSS containing 60 mM K^+^. Cells were then held at −50 mV and subjected to 200 ms voltage ramps from −120 mV to +20 mV in the absence or presence of Ba^2+^ (100 µM). Currents were normalized to cell capacitance.

Samples were prepared using a modified isolation protocol for single-cell qRTPCR, as described.^25,26^ Briefly, SEA SMCs were enzymatically isolated, and samples of ∼50 cells were aspirated into glass micropipettes filled with RNA isolation buffer for use in Ambion® Single Cell-to-C_T_ qRTPCR kit (ThermoFisher Scientific). Reactions were then prepared using TaqMan gene expression MasterMix (ThermoFisher Scientific) and primers for K_IR_2.1 (*Kcnj2*; RefSeq NM_008425.4), K_IR_2.2 (*Kcnj12*; RefSeq NM_001267593.1) and smooth muscle α-actin (*Acta2*; RefSeq NM_007392.3) (ThermoFisher Scientific). Transcripts were preamplified for 14 cycles prior to qRTPCR, as per manufacturer’s instructions. Quantitative RTPCR for the transcripts of interest was then run for 40 cycles. No-template controls were included throughout. Sample mRNA expression of *Kcnj2* and *Kcnj12* were normalized to *Acta2* expression as described.^25^

### Superior epigastric vessel myography

SEAs were cannulated onto glass micropipettes and studied by pressure myography as described.^22–24^ Vessels were warmed to 37 °C, pressurized to 80 cm H_2_O and allowed to develop myogenic tone or were pre-constricted with phenylephrine if myogenic tone was less than 10% (10^−6^ M). The cannulated arteries were superfused with PSS (5 mM K^+^), or solutions containing 8 mM or 15 mM K^+^ (KCl substituted for NaCl in PSS) in the absence or presence of Ba^2+^ (100 µM).

### Statistical analysis

Data were analyzed using GraphPad Prism 8 (GraphPad Software) and are expressed as mean ± SD. Distribution was determined with Shapiro-Wilk test. P < 0.05 was considered statistically significant.

## Results

### K_IR_ currents persist despite K_IR_2.1 ablation in smooth muscle cells of cerebral arteries

**Figure 1.**
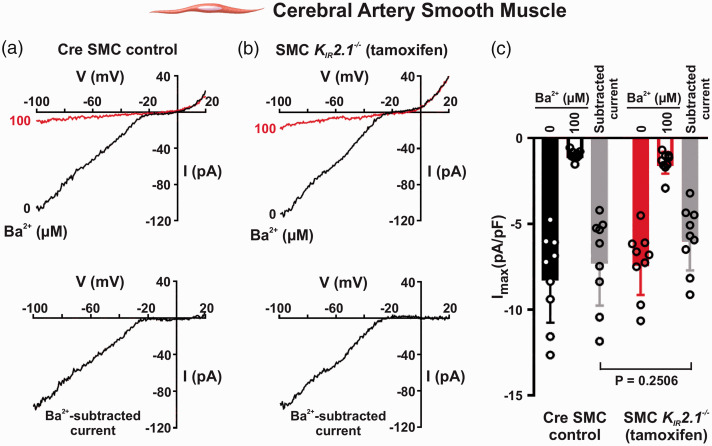
Genetic ablation of K_IR_2.1 does not eliminate inward K^+^ currents in cerebral arterial smooth muscle cells (SMCs). Whole-cell patch clamp electrophysiology was used to measure K_IR_ current with voltage ramps from −100 to +20 mV in the absence and presence of Ba^2+^ in 60 mM K^+^. (a,b) Representative recordings of whole-cell and Ba^2+^-subtracted K_IR_ currents in myocytes isolated from SMC *K_IR_2.1^−/−^* mice and non-induced Cre SMC controls. (c) Summary data compare peak inward current at −100 mV between groups (*n* = 9 SMCs from 6 mice in control group and *n* = 9 SMCs from 8 mice in knockout group; nested *t*-test).

In cerebral arterial smooth muscle, K_IR_2.1 subunits are presumed to be the dominant isoform driving the native K_IR_2.x current.^10^ To explore this concept, initial experiments sought to delineate the impact of K_IR_2.1 knockout on whole-cell Ba^2+^-sensitive inwardly rectifying K^+^ currents in freshly isolated SMCs. Figure 1 denotes the presence of a robust inward current in both tamoxifen-induced SMC *K_IR_2.1^−/−^* and non-induced control animals (Cre recombinase carriers with floxed K_IR_2.1 gene). The observed currents were readily activated by extracellular K^+^ and blocked by micromolar Ba^2+^, signature characteristics of vascular K_IR_2.x channels. On aggregate, Ba^2+^-sensitive currents were modestly reduced in SMC *K_IR_2.1^−/−^* mice (5.83 ± 1.88 pA/pF) compared to controls (7.12 ± 2.63 pA/pF), but this difference was not statistically significant (Figure 1(c)). This absence of K_IR_ current knockdown was also observed in SMCs isolated from superior epigastric arteries (SEAs) of SMC *K_IR_2.1^−/−^* mice (Figure S2(a,b)). To verify that Cre SMC control mice do not have reduced K_IR_ current potentially resulting from leaky Cre recombinase activity, Ba^2+^-sensitive K^+^ currents were measured in cerebral SMCs of C57BL/6 mice. We found no difference between non-induced Cre SMC control and C57BL/6 control animals (7.12 ± 2.63 pA/pF vs. 6.88 ± 1.67 pA/pF respectively, P = 0.85, unpaired *t*-test).

### K_IR_2.1 expression was reduced post-knockout in cerebral arterial smooth muscle cells without impact on K_IR_2.2

**Figure 2.**
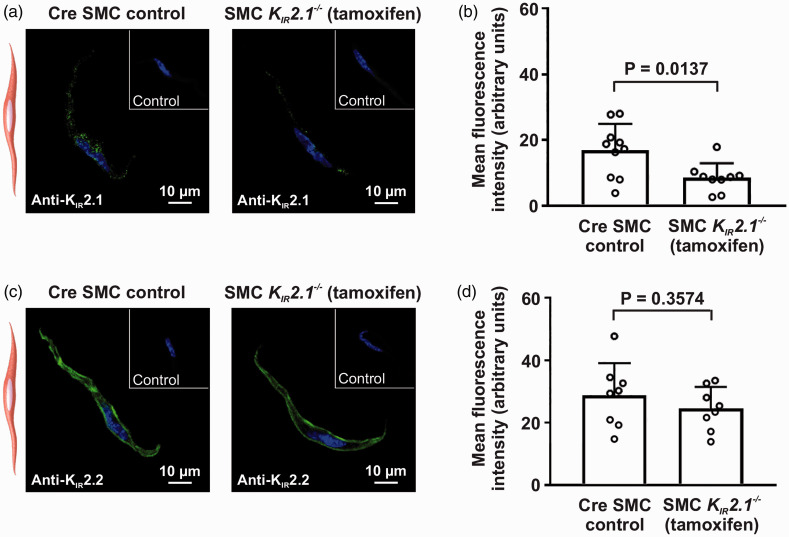
K_I__R_2.1 is negligibly expressed in cerebral vascular smooth muscle cells (SMCs) while K_IR_2.2 is highly expressed at the cell membrane. Tamoxifen-induced K_IR_2.1 knockout significantly reduced subunit expression but levels remained detectable by immunofluorescence. (a) Fluorescent anti-K_IR_2.1 (green) exhibited a faint labeling pattern in cerebral arterial myocytes from SMC *K_IR_2.1^−/−^* and control mice with nuclei stained with DAPI (blue). (b) Summary of data compares fluorescence intensity (background subtracted) of K_IR_2.1 signal between groups (*n* = 10 cells from 5 animals in control group and *n* = 9 cells from 5 animals in knockout group; unpaired *t*-test). (c) Immunofluorescence labeling of SMCs for K_IR_2.2. (d) Summary of data compares background-subtracted fluorescence intensity of K_IR_2.2 signal between groups (*n* = 8 cells pooled from 4 animals/group; unpaired *t*-test). Two cells were analyzed per animal with background signal subtracted using 2° antibody control.

**Figure 3.**
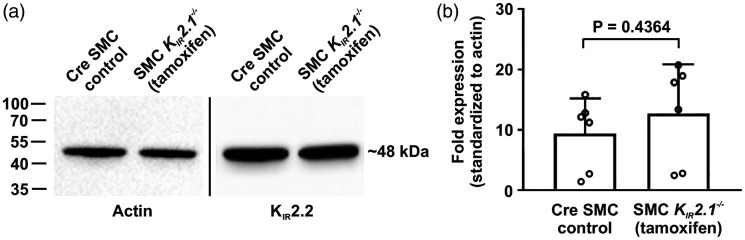
K_IR_2.2 protein expression is unaltered in smooth muscle cell (SMC) *K_IR_2.1^−/−^* cerebral arteries. (a) Western blot of intact cerebral arteries confirmed that protein levels of K_IR_2.2 are not impacted by deletion of SMC K_IR_2.1. (b) Summary of data compares K_IR_2.2 protein levels between control and knockout mice (normalized to actin; *n* = 6 mice; unpaired *t*-test).

The whole-cell currents we observed, while indicative of strong rectifying K_IR_2.x activity, likely exist as a composite of all K_IR_2.x channels expressed in VSM. Therefore, to further discern the contributions of individual K_IR_2.x subunits, we screened for subunits commonly described in cerebral arterial smooth muscle—K_IR_2.1 and K_IR_2.2.^15^ Immunostaining of K_IR_2.1 revealed a diffuse expression pattern (Figure 2(a)). Mean global cell fluorescence of K_IR_2.1 antibody signal was reduced in SMC *K_IR_2.1^−/−^* mice when compared with the control group (Figure 2(b)). In contrast, K_IR_2.2 was predominantly concentrated at the plasma membrane (Figure 2(c)) and the induction of K_IR_2.1 deletion did not impact the expression of K_IR_2.2 (Figure 2(d)). To further confirm that SMC K_IR_2.1 deletion does not lead to compensatory changes in K_IR_2.2 expression, western blot analysis was performed for K_IR_2.2. Since K_IR_2.2 is expected to be expressed in SMCs and not endothelial cells (ECs), intact cerebral arteries were used. Indeed, protein levels of K_IR_2.2 were similar in control and SMC *K_IR_2.1^−/−^* mice (Figure 3).

**Figure 4.**
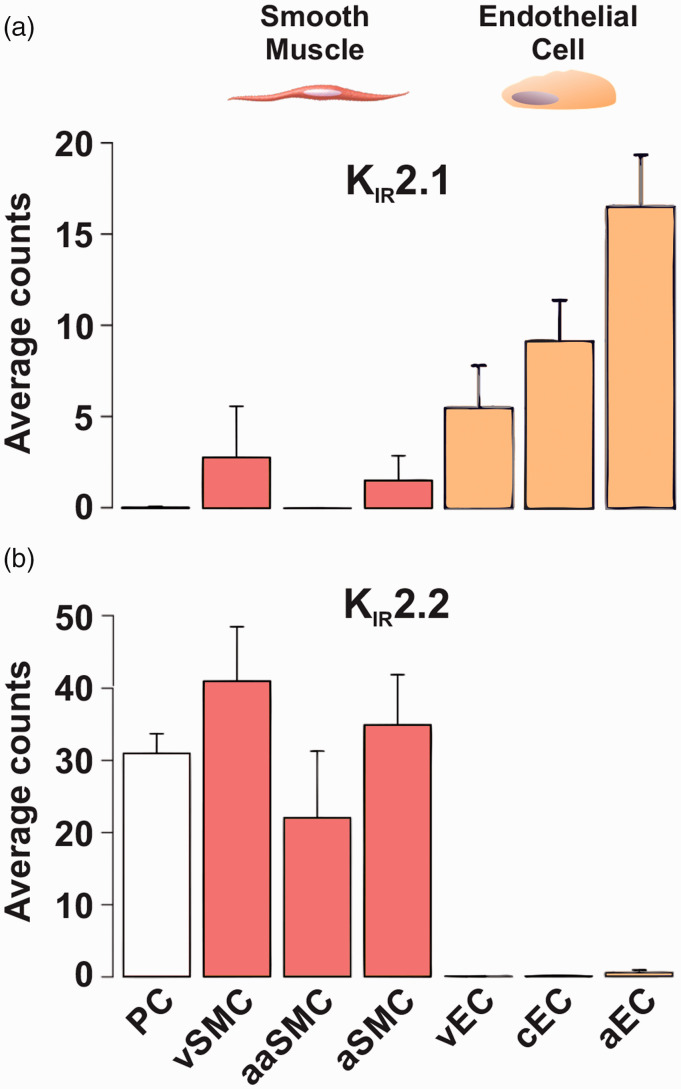
K_IR_2.1 and K_IR_2.2 subunits are inversely expressed in cerebral endothelial and vascular smooth muscle cells. (a) K_IR_2.1 and (b) K_IR_2.2 subunit expression is shown as average cellular transcript counts per cell, as determined by single-cell RNA sequencing of the mouse brain vasculature. Data highlight differences in the dominant subunit between cell types. Abbreviations: PC – pericytes; SMC – smooth muscle cells; EC – endothelial cells; v – venous; c – capillary; a – arterial; aa – arteriolar. Figures provided by http://betsholtzlab.org/VascularSingleCells/database.html.^33,34^

While contrary to initial reports,^10,17^ the expression pattern in our study aligns with more recent molecular work highlighting K_IR_2.2 dominance over K_IR_2.1 at the mRNA level in mouse cerebral arterial SMCs (Figure 4). Furthermore, Q-PCR assessments performed on SMCs from SEAs revealed a comparable expression pattern, with K_IR_2.2 dominating over K_IR_2.1 (Figure S2(c)).

### Myogenic responses and K^+^-induced dilation are maintained in cerebral arteries of SMC K_IR_2.1^−/−^ mice

**Figure 5.**
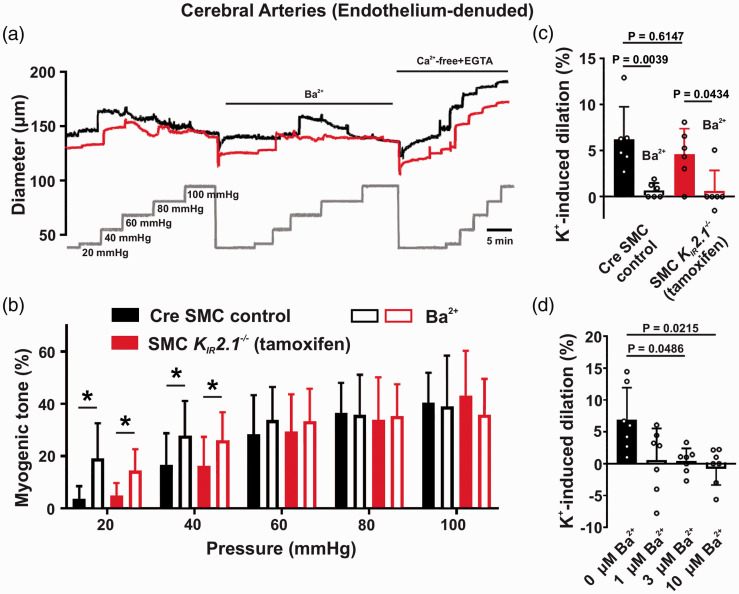
Myogenic responses and K^+^-induced dilation are intact in cerebral arteries of smooth muscle cell (SMC) *K_IR_2.1^−/−^* mice. Cerebral arteries from control and SMC *K_IR_2.1^−/−^* mice were cannulated and intravascular pressure was elevated stepwise while vasomotor responses were measured. (a) Representative diameter traces from endothelium-denuded vessels of control and SMC *K_IR_2.1^−/−^* mice show the effect of increasing pressure on myogenic tone. (b) Summary of data highlights limited impact of smooth muscle K_IR_2.1 knockout on myogenic tone development. Paired *t*-test was performed for 0 vs. 100 μM Ba^2+^ treatment (*n* = 10 vessels in the control group and *n* = 11 vessels in the SMC *K_IR_2.1^−/−^* group with 1 vessel/mouse; *P < 0.05). (c) K^+^-induced dilation, elicited by increasing extracellular K^+^ from 5 mM to 10 mM before and after treatment with 100-μM Ba^2+^, was intact in the knockout group (*n* = 6 vessels in the control group and SMC *K_IR_2.1^−/−^* group; one-way ANOVA with Sidak’s multiple comparisons test). (d) K^+^-induced dilation (5 mM K^+^ to 10 mM K^+^) was abrogated with exposure to low concentrations of Ba^2+^, implicating K_IR_2.2 as the mediator of this response based on its Ba^2+^ sensitivity profile (*n* = 7 vessels from control mice; repeated measures one-way ANOVA with Sidak’s multiple comparisons test). Myogenic tone (%) was calculated as: [(passive diameter – active diameter)/(passive diameter – minimal diameter)] × 100 at each pressure step. K^+^-induced dilation (%) was calculated as difference between diameter at 10 mM [K^+^] and 5 mM [K^+^] divided by the dilatory range (passive diameter – minimal diameter).

K_IR_ channels maintain resistance arteries in a hyperpolarized state at low pressure and their activity diminishes as vessels develop tone at higher pressure.^15^ We examined the impact of SMC K_IR_2.1 deletion on myogenic tone development in endothelium-denuded cerebral arteries. Lack of dilatory response to bradykinin confirmed successful disruption of endothelium; 144 ± 16 μm vs. 144 ± 15 μm diameter before and after application of bradykinin, respectively, *n* = 11. As control arteries were pressurized from 20–100 mmHg, a strong pressure-induced constriction was observed, a response that was comparable to vessels isolated from tamoxifen-induced mice (not statistically significant based on an unpaired *t*-test; Figure 5(a) and (b)). To discern K_IR_ activity, Ba^2+^ was added to bath solution. At low pressures (20–40 mmHg), where vessels are hyperpolarized with low myogenic tone, Ba^2+^ treatment induced constriction (Figure 5(b)). As pressure was elevated and vessels depolarized, the effects of Ba^2+^ became attenuated. This response pattern was comparable between the two groups, indicating that K_IR_ contributions to the functional phenotype of the de-endothelialized vessels were intact despite SMC-specific K_IR_2.1 deletion. Increasing extracellular K^+^ potentiates K_IR_ channels, promoting membrane hyperpolarization and vessel dilation.^27^ Bath K^+^ was increased from 5 mM to 10 mM and dilatory responses attributed to K_IR_ were measured before and after treatment with 100-μM Ba^2+^. This experiment further confirmed that SMC K_IR_2.1 knockout had no significant functional effect as K^+^-induced dilation was present in SMC *K_IR_2.1^−/−^* vessels and was abrogated after treatment with 100-μM Ba^2+^ in both groups (Figure 5(c)). Specifically, Ba^2+^-sensitive, K^+^-induced vasodilation was 5.5 ± 3.9% in control vessels and 4.0 ± 2.3% in vessels from SMC *K_IR_2.1^−/−^* mice. Intriguingly, this response was also significantly blocked at low Ba^2+^ concentrations (3 μM; Figure 5(d)), a finding more consistent with the Ba^2+^ sensitivity profile of K_IR_2.2.^16^ We also examined dilatory responses in pressurized SEAs and found that Ba^2+^-sensitive, K^+^-induced vasodilation was comparable between the two groups (Figure S2(e)).

### Region-specific brain perfusion is maintained in SMC K_IR_2.1^−/−^ mice

**Figure 6.**
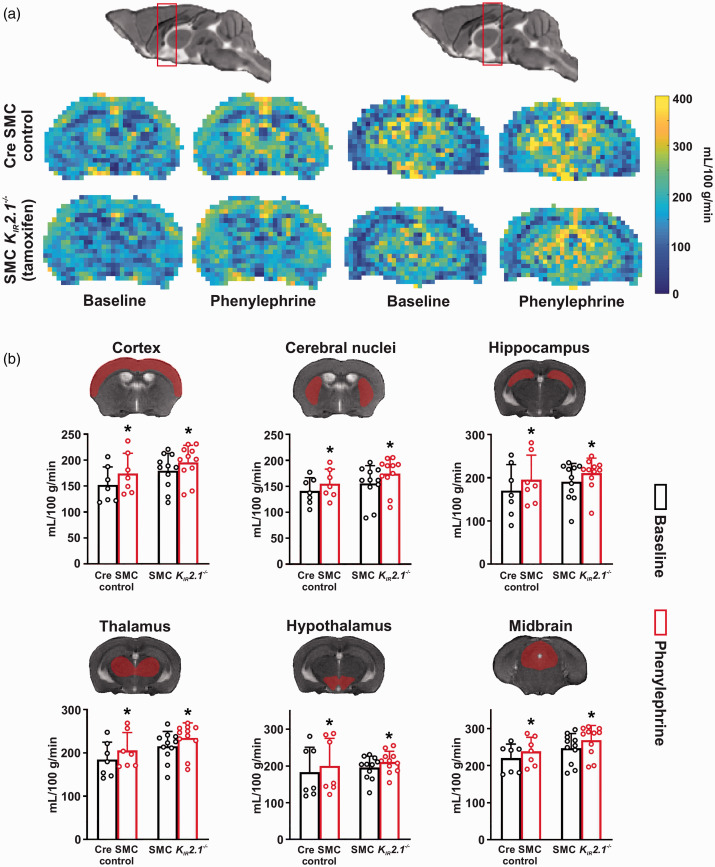
Region-specific brain perfusion is not altered in smooth muscle cell (SMC) *K_IR_2.1^−/−^* mice at rest and with increased systemic blood pressure. (a) Representative arterial spin-labeled MR brain perfusion maps. Scans were done in a posterior-to-anterior direction and the volume of brain scanned was divided into 5 coronal slices. Resting cerebral blood flow was measured in control and SMC *K_IR_2.1^−/−^* mice. Scans were repeated after blood pressure challenge with an intraperitoneal phenylephrine injection. Figure shows slices from 2 regions of the brain (red boxes) spanning cerebral nuclei, hippocampus, thalamus, and hypothalamus. (b) Baseline perfusion in several major brain structures was not significantly different between control and tamoxifen-induced mice. The blood pressure challenge caused a modest but significant rise in cerebral blood flow to a similar extent in control and SMC *K_IR_2.1^−/−^* animals. Unpaired *t*-test was performed for control (*n* = 7 mice) vs. SMC *K_IR_2.1^−/−^* (*n* = 11 mice) comparison; paired *t*-test was performed for baseline vs. phenylephrine-treatment. *P < 0.05 compared to baseline control.

Functional hyperemia in the brain, often termed neurovascular coupling, has been linked to the activation of SMC K_IR_ by extracellular K^+^.^28^ These channels have also been implicated in cerebral autoregulation as they respond to changes in intravascular pressure.^15^ Considering these functions, we assessed the effects of SMC-specific K_IR_2.1 knockout on cerebral perfusion at rest and in response to a systemic blood pressure challenge. Animals were instrumented and placed in an MRI scanner for blood flow measurement by ASL before and after intraperitoneal phenylephrine injection. At rest, there were no significant differences between control and SMC *K_IR_2.1^−/−^* mice in perfusion of the cortex, cerebral nuclei, hippocampus, thalamus, hypothalamus, and midbrain (Figure 6). Phenylephrine injection increased MAP from 99 ± 16 mmHg to 109 ± 17 mmHg (P < 0.05), as measured via tail cuff. Of note, MAP did not differ between the two groups of mice and so results were pooled. The phenylephrine treatment resulted in a small but significant rise in CBF in all the brain regions analyzed (Figure 6). However, the magnitude of this response did not differ between the two groups (Figure 6(b)).

### Knockout of SMC K_IR_2.1 has no significant effect on Ba^2+^-sensitive currents and K^+^-induced dilation of superior epigastric arteries

In SEAs, tamoxifen-inducible knockout of SMC K_IR_2.1 had no significant effect on Ba^2+^-sensitive currents compared to control C57BL/6 mice (Figure S2(a,b)). In fact, mRNA analysis indicated that K_IR_2.2 expression dominates K_IR_2.1 expression in epigastric artery SMCs of SMC *K_IR_2.1^−/−^* mice (Figure S2(c)) and C57BL/6 control mice, as we previously showed.^23^ We also examined myogenic tone in these vessels under pressure (Figure S2(d)). While Ba^2+^-induced constriction in 5 mM K^+^ PSS was similar in C57BL/6 (20 ± 3.4% constriction, *n* = 5) and SMC *K_IR_2.1^−/−^* mice (17 ± 18% constriction, *n* = 8; P = 0.7241; Mann-Whitney U-test), myogenic tone at 80 cm H_2_O was significantly increased in the knockout with considerable variability. Elevated extracellular K^+^ dilated SEAs from C57BL/6 mice in a concentration-dependent manner and Ba^2+^ (100 μM) inhibited this response, implicating K_IR_ channels in the mechanism of action (Figure S2(e)). Knockout of SMC K_IR_2.1 had no effect on the magnitude of this dilatory response.

## Discussion

This study explored the role that K_IR_2.1 plays within the cerebral vasculature using an inducible, SMC-specific genetic ablation model. The persistence of Ba^2+^-sensitive K^+^ currents following deletion of K_IR_2.1 raised the possibility that other subunits underlie the smooth muscle inward K^+^ conductance. Functional experiments reinforced this idea as reduced SMC expression of K_IR_2.1 did not markedly impact myogenic reactivity of cerebral arteries and maintenance of blood flow to various brain regions. The high expression of K_IR_2.2 subunits, as detected by immunofluorescence and RNA sequencing in myocytes, may account for this minimal functional impact. The lack of phenotype following K_IR_2.1 knockout was mirrored in experiments conducted in SEAs, indicating that these observations are not limited to the cerebral vasculature. These findings highlight that arterial responses are not dependent on K_IR_2.1 expression in SMCs and suggest the involvement of a more diverse pool of K_IR_ subunits.

Contractility of the VSM layer is tightly coupled to intracellular Ca^2+^ sourced through VGCCs, thereby creating a close relationship between V_M_ and diameter.^7^ K^+^ channels regulate the influx of Ca^2+^ by hyperpolarizing V_M_ to promote relaxation and vasodilation.^8^ The strong-rectifying K_IR_2.x family of channels are especially prominent within cerebral vessels where they are active under basal conditions to hyperpolarize V_M_.^8,15,29^ Although their role appears subtle, altering K_IR_ activity translates to significant effects on vessel diameter and reactivity. K_IR_ channel inhibition with Ba^2+^ causes vasoconstriction, reduces flow, and prevents K^+^-induced dilation in rat cerebral and human brachial arteries.^3,14,30^ Furthermore, K_IR_ channels amplify other dilatory responses through their intrinsic property of negative slope conductance.^19,29,31^ They are also modulated by hemodynamic forces, specifically intravascular pressure and flow.^14,15^ Given their pleiotropic roles, it is logical to expect that knockout would generate a robust physiological phenotype.

This study began by examining native K_IR_ currents in vascular smooth muscle isolated from cerebral arteries. To our surprise, comparable K_IR_ activity was detected in SMC *K_IR_2.1^−/−^* and control mice. While currents trended slightly lower in the knockout animals, no significant difference was observed between the two groups. These findings suggest the predominance of another K_IR_ subunit—one with similar electrophysiological properties. Based on Ba^2+^ sensitivity and unitary conductance, K_IR_2.2 is a logical target and it should be noted that this subunit can heteromultimerize with K_IR_2.1.^16,32^ With this in mind, we sought to observe the localization of key K_IR_ subunits in cerebral arterial SMCs. Antibodies revealed a punctate labeling pattern for K_IR_2.1 that was weak in control mice and was further diminished after tamoxifen-induced conditional deletion. As to other pertinent K_IR_ subunits, we found that K_IR_2.2 is abundantly concentrated at the SMC plasma membrane. While these results contradict the historical view of K_IR_2.1 being the dominant subtype in cerebral SMCs, they do align with recent reports on single-cell RNA sequencing of cerebrovascular cells.^33,34^ Specifically, this transcriptomic analysis revealed that K_IR_2.1, while being highly expressed in ECs, was decidedly less abundant in arterial SMCs.^33,34^ Conversely, K_IR_2.2 mRNA expression was markedly greater in SMCs but negligible in ECs. A similar pattern was reported in SMCs and ECs from SEAs.^23^ This differential expression pattern, in concert with our functional data, reinforces the notion that expression of K_IR_2.1 in SMCs is not central to cerebral vessel function.

Elevation of extracellular K^+^ enhances K_IR_ channel activity and drives arterial dilation. This stimulus is thought to contribute to functional hyperemia in the brain.^28^ In our SMC *K_IR_2.1^−/−^* mice, Ba^2+^-sensitive K^+^-induced dilation was conserved and equal in magnitude to control animals. Thus, while our data show this response is K_IR_ mediated, another subtype appears to be involved. Based on expression data,^33,34^ K_IR_2.2 was viewed as the most likely subtype. The identity of K_IR_ subunits driving cell-specific K_IR_ currents can be differentiated based on their Ba^2+^ sensitivity; K_IR_2.1 and K_IR_2.2 both are blocked in the micromolar range but K_IR_2.2 is 10 times more sensitive.^16,32^ Thus, we tested K^+^-induced dilation in cerebral arteries of control mice before and after application of low concentrations of Ba^2+^ (1 μM to 10 μM) and found this response was abolished, consistent with K_IR_2.2 activity.

Our electrophysiology and myography results contrast work in a global *K_IR_2.1^−/−^* mouse,^17^ where genetic deletion abolished inward K^+^ currents in cerebral SMCs and K^+^-induced dilation. While seemingly at odds, it should be recognized that the global knockout displays severe developmental defects (cleft palate) and as death occurs within 24 hrs of birth, neonatal rather than adult vessels were used—a technically challenging prospect. This can be problematic as early postnatal SMCs may not be fully differentiated, differing in contractile phenotype compared to adult SMCs.^35–37^ It should also be noted that K_IR_2.1 is functionally important in cerebral arterial ECs, and, as such, the functional phenotype changes observed in the global knockout may be influenced by the deletion in ECs.

Smooth muscle K_IR_ channels are sensitive to hemodynamic forces; as intraluminal pressure rises, their activity is suppressed whereas low pressure has the opposite effect.^14,15^ This aligns with increased Ba^2+^ sensitivity—reflective of greater K_IR_ activity—of cerebral arteries at lower pressures.^15^ Intriguingly, pressure sensitivity was reported to be modulated by cholesterol, with depletion increasing K_IR_ activity in SMCs, consistent with this lipid stabilizing K_IR_ channels in the closed state.^15^ This finding translated into whole vessels, with increased Ba^2+^-induced constriction being observed in denuded cerebral arteries at elevated pressure after cholesterol depletion.^15^ This work helped define a new paradigm of hemodynamic sensing coupled to specific K_IR_-lipid interactions in the cerebral vasculature. Based on the pressure-K_IR_ relationship, we expected mice deficient in smooth muscle K_IR_2.1 to exhibit diminished myogenic responses to pressure. Such an impairment would be expected to impact CBF regulation, manifesting as enhanced brain perfusion on ASL perfusion maps. Contrary to these expectations, we found that region-specific brain perfusion was comparable between SMC *K_IR_2.1^−/−^* mice and controls and did not differ among the two groups after a phenylephrine-induced systemic blood pressure challenge. This implies a limited role for SMC K_IR_2.1 in conferring pressure sensitivity in the cerebral vasculature. One limitation of our study is that CFB was not fully maintained at baseline after high dose phenylephrine, perhaps due to blunting of autoregulation by isoflurane.^38^ Tail cuff measure of blood pressure accentuates this concern because it underestimates true in vivo readings.^39^ As such, our phenylephrine challenge likely elicited a response greater than 10 mmHg, which also implies tone was not heavily impaired. Finally, it should be noted that complexity of the MRI setup precluded collection of end-tidal CO_2_ values during the scans, although we did actively monitor and maintain breath rate with small anesthetic flow adjustments.

K_IR_ currents in ECs are markedly larger than those in SMCs. K_IR_2.1 is indispensable in ECs with a major role in endothelium-dependent dilation, particularly in amplifying the impact of other active vasodilatory K^+^ channels.^19^ Endothelial K_IR_ channels are also tied to hemodynamic sensing, with shear stress potentiating the native cerebral arterial current. This potentiation facilitated flow-induced vasodilation and was dependent on interactions with phosphatidylinositol 4,5-bisphosphate.^15^ The ability of lipids to regulate EC and SMC K_IR_ highlights the potential involvement of these channels in pathophysiological mechanisms in dyslipidemic disorders.

Based on our work, we believe that the observed differences between EC and SMC K_IR_ channel pools in hemodynamic and lipid sensitivity stem from differences in subunit composition. Subunit identity further becomes relevant when considering molecular interactions in mechanotransduction pathways potentially involving K_IR_2.x subunits. Specifically, K_IR_2.1 and K_IR_2.2 differ in protein binding capability, which may in turn influence the formation of signalling complexes. Moreover, several members of the dystrophin-associated protein complex, which links the plasma membrane to the cytoskeleton, associate more strongly with the C-terminus of K_IR_2.2 as opposed to K_IR_2.1 where interactions are weak.^40^ This disparity was attributed to differences in PDZ binding motifs and upstream amino acid sequences of these channels. While the differences are intriguing, functional implications of these interactions remain unexplored.

We also examined the role of K_IR_2.1 in a skeletal muscle resistance artery—the superior epigastric artery. K_IR_ channels appear to contribute substantially to resting membrane potential and myogenic tone at normal blood pressure levels in SEAs.^23^ We previously reported that SMCs in these arteries predominately express mRNA for K_IR_2.2 with little mRNA for K_IR_2.1 in C57BL/6 mice.^23^ Consistent with these findings, we observed low abundance of K_IR_2.1 in superior epigastric myocytes from SMC *K_IR_2.1^−/−^* mice, whereas K_IR_2.2 expression was substantially higher and similar to reported levels for this arterial network.^23^ SMC K_IR_ currents and Ba^2+^-sensitive, K^+^-induced dilation of isolated SEAs appeared unaffected by deletion of SMC K_IR_2.1. This suggests, similar to cerebral vessels, SMC K_IR_2.1 does not play a major role. However, we did observe increased myogenic tone in isolated SEAs in our knockout, indicating that there may be some subtle effect of SMC K_IR_2.1 deletion in these vessels. The mechanism responsible for this elevated myogenic tone was not explored further. Nonetheless, all other measures of K_IR_ channel function, including K_IR_ currents, K^+^-induced vasodilation, and Ba^2+^-induced constriction, appeared unaffected by knockout of K_IR_2.1 in SMCs. These data lead us to conclude, that, like the cerebral vasculature, expression and function of K_IR_2.1 channels in SEA SMCs appears minimal, and other K_IR_ channels such as K_IR_2.2 may be more functionally relevant.

## Summary

Cerebral blood flow is a highly regulated process, where adjustments to perfusion occur in response to transient local stimuli and sustained hemodynamic forces. These adjustments ensure adequate nutrient delivery while preventing brain injury from hypo- or hypertension. This remarkable feat is intimately tied to a diverse collection of ion channels in ECs and SMCs whose composite actions regulate vascular tone. Expressed in both ECs and SMCs, K_IR_ channels participate in tone regulation though V_M_ establishment, K^+^ response amplification, and force sensing in cerebral arteries.^3,15,19,29^ Historically, K_IR_2.1 was viewed as a major contributor to the inward K^+^ conductance in cerebral SMCs. Our observations lie in contrast, with K_IR_ activity persisting at the isolated cell, intact vessel, and live animal levels after SMC-specific K_IR_2.1 knockout. This minimal functional impact suggests that K_IR_2.1 is either a redundant component of the channel pool or its activity is minor relative to other K_IR_ subunits in VSM. Indeed, we found that K_IR_2.1 expression in SMCs is modest, while a different subunit—K_IR_2.2—was found abundantly localized to the plasma membrane. To conclude, our work highlights that cerebral SMC K_IR_ responses are not dependent on K_IR_2.1 and suggests that the native channel pool is more heterogenous in composition than previously thought.

## Supplemental Material

sj-pdf-1-jcb-10.1177_0271678X221093432 - Supplemental material for Genetic ablation of smooth muscle K_IR_2.1 is inconsequential to the function of mouse cerebral arteries Click here for additional data file. Supplemental material, sj-pdf-1-jcb-10.1177_0271678X221093432 for Genetic ablation of smooth muscle K_IR_2.1 is inconsequential to the function of mouse cerebral arteries by Paulina M Kowalewska, Jacob Fletcher, William F Jackson, Suzanne E Brett, Michelle SM Kim, Galina Yu Mironova, Nadia Haghbin, David M Richter, Nathan R Tykocki, Mark T Nelson and Donald G Welsh in Journal of Cerebral Blood Flow & Metabolism

sj-pdf-2-jcb-10.1177_0271678X221093432 - Supplemental material for Genetic ablation of smooth muscle K_IR_2.1 is inconsequential to the function of mouse cerebral arteries Click here for additional data file. Supplemental material, sj-pdf-2-jcb-10.1177_0271678X221093432 for Genetic ablation of smooth muscle K_IR_2.1 is inconsequential to the function of mouse cerebral arteries by Paulina M Kowalewska, Jacob Fletcher, William F Jackson, Suzanne E Brett, Michelle SM Kim, Galina Yu Mironova, Nadia Haghbin, David M Richter, Nathan R Tykocki, Mark T Nelson and Donald G Welsh in Journal of Cerebral Blood Flow & Metabolism

## Data Availability

The data that support the findings of the study are included in this manuscript.

## References

[1] Segal SS Duling BR. Flow control among microvessels coordinated by intercellular conduction. Science 1986; 234: 868–870. 3775368 10.1126/science.3775368

[2] Zechariah A Tran CHT Hald BO , et al. Intercellular conduction optimizes arterial network function and conserves blood flow homeostasis during cerebrovascular challenges. Arterioscler Thromb Vasc Biol 2020; 40: 733–750. 31826653 10.1161/ATVBAHA.119.313391 PMC7058668

[3] Knot HJ Zimmermann PA Nelson MT. Extracellular K^+^-induced hyperpolarizations and dilatations of rat coronary and cerebral arteries involve inward rectifier K^+^ channels. J Physiol 1996; 492: 419–430. 9019539 10.1113/jphysiol.1996.sp021318 PMC1158837

[4] Knot HJ Nelson MT. Regulation of arterial diameter and wall [Ca^2+^] in cerebral arteries of rat by membrane potential and intravascular pressure. J Physiol 1998; 508: 199–209. 9490839 10.1111/j.1469-7793.1998.199br.x PMC2230857

[5] Zhang R Zuckerman JH Iwasaki K , et al. Autonomic neural control of dynamic cerebral autoregulation in humans. Circulation 2002; 106: 1814–1820. 12356635 10.1161/01.cir.0000031798.07790.fe

[6] Cole WC Welsh DG. Role of myosin light chain kinase and myosin light chain phosphatase in the resistance arterial myogenic response to intravascular pressure. Arch Biochem Biophys 2011; 510: 160–173. 21392499 10.1016/j.abb.2011.02.024

[7] Thorneloe KS Nelson MT. Ion channels in smooth muscle: regulators of intracellular calcium and contractility. Can J Physiol Pharmacol 2005; 83: 215–242. 15870837 10.1139/y05-016

[8] Nelson MT Quayle JM. Physiological roles and properties of potassium channels in arterial smooth muscle. Am J Physiol Physiol 1995; 268: C799–C822. 10.1152/ajpcell.1995.268.4.C799 7733230

[9] Quayle JM Nelson MT Standen NB. ATP-sensitive and inwardly rectifying potassium channels in smooth muscle. Physiol Rev 1997; 77: 1165–1232. 9354814 10.1152/physrev.1997.77.4.1165

[10] Bradley KK Jaggar JH Bonev AD , et al. Kir2.1 encodes the inward rectifier potassium channel in rat arterial smooth muscle cells. J Physiol 1999; 515: 639–651. 10066894 10.1111/j.1469-7793.1999.639ab.x PMC2269194

[11] Hibino H Inanobe A Furutani K , et al. Inwardly rectifying potassium channels: their structure, function, and physiological roles. Physiol Rev 2010; 90: 291–366. 20086079 10.1152/physrev.00021.2009

[12] Quayle JM McCarron JG Brayden JE , et al. Inward rectifier K^+^ currents in smooth muscle cells from rat resistance-sized cerebral arteries. Am J Physiol Physiol 1993; 265: C1363–C1370. 10.1152/ajpcell.1993.265.5.C1363 7694496

[13] Shieh R-C Chang J-C Arreola J. J. Interaction of Ba^2+^ with the pores of the cloned inward rectifier K^+^ channels Kir2.1 expressed in *xenopus* oocytes. Biophys J 1998; 75: 2313–2322. 9788926 10.1016/S0006-3495(98)77675-1 PMC1299905

[14] Wu B-N Luykenaar KD Brayden JE , et al. Hyposmotic challenge inhibits inward rectifying K^+^ channels in cerebral arterial smooth muscle cells. Am J Physiol Heart Circ Physiol 2007; 292: H1085–H1094. 17056667 10.1152/ajpheart.00926.2006

[15] Sancho M Fabris S Hald BO , et al. Membrane lipid-K_IR_2.x channel interactions enable hemodynamic sensing in cerebral arteries. Arterioscler Thromb Vasc Biol 2019; 39: 1072–1087. 31043073 10.1161/ATVBAHA.119.312493

[16] Schram G Pourrier M Wang Z , et al. Barium block of Kir2 and human cardiac inward rectifier currents: evidence for subunit-heteromeric contribution to native currents. Cardiovasc Res 2003; 59: 328–338. 12909316 10.1016/s0008-6363(03)00366-3

[17] Zaritsky JJ Eckman DM Wellman GC , et al. Targeted disruption of Kir2.1 and Kir2.2 genes reveals the essential role of the inwardly rectifying K^+^ current in K^+^-mediated vasodilation. Circ Res 2000; 87: 160–166. 10904001 10.1161/01.res.87.2.160

[18] Wirth A Benyó Z Lukasova M , et al. G12-G13–LARG–mediated signaling in vascular smooth muscle is required for salt-induced hypertension. Nat Med 2008; 14: 64–68. 18084302 10.1038/nm1666

[19] Sonkusare SK Dalsgaard T Bonev AD , et al. Inward rectifier potassium (Kir2.1) channels as end-stage boosters of endothelium-dependent vasodilators. J Physiol 2016; 594: 3271–3285. 26840527 10.1113/JP271652 PMC4908010

[20] Kilkenny C Browne WJ Cuthill IC , et al. Improving bioscience research reporting: the ARRIVE guidelines for reporting animal research. PLoS Biol 2010; 8: e1000412. 20613859 10.1371/journal.pbio.1000412 PMC2893951

[21] Nieves-Cintrón M Tajada S Santana LF , et al. Total internal reflection fluorescence microscopy in vascular smooth muscle. Signal Transduct Smooth Muscle 2018; 5: 87–103.

[22] Hayoz S Bradley V Boerman EM , et al. Aging increases capacitance and spontaneous transient outward current amplitude of smooth muscle cells from murine superior epigastric arteries. Am J Physiol Circ Physiol 2014; 306: H1512–H1524. 10.1152/ajpheart.00492.2013 PMC4042199 24705555

[23] Hayoz S Pettis J Bradley V , et al. Increased amplitude of inward rectifier K^+^ currents with advanced age in smooth muscle cells of murine superior epigastric arteries. Am J Physiol Circ Physiol 2017; 312: H1203–H1214. 10.1152/ajpheart.00679.2016 PMC6146378 28432059

[24] Mullan B Pettis J Jackson WF. T-type voltage-gated Ca^2+^ channels do not contribute to the negative feedback regulation of myogenic tone in murine superior epigastric arteries. Pharmacol Res Perspect 2017; 5: e00320. 28603637 10.1002/prp2.320 PMC5464347

[25] Westcott EB Goodwin EL Segal SS , et al. Function and expression of ryanodine receptors and inositol 1,4,5-trisphosphate receptors in smooth muscle cells of murine feed arteries and arterioles. J Physiol 2012; 590: 1849–1869. 22331418 10.1113/jphysiol.2011.222083 PMC3573308

[26] Longden TA Dabertrand F Koide M , et al. Capillary K^+^-sensing initiates retrograde hyperpolarization to increase local cerebral blood flow. Nat Neurosci 2017; 20: 717–726. 28319610 10.1038/nn.4533 PMC5404963

[27] Burns WR Cohen KD Jackson WF. K^+^-induced dilation of hamster cremasteric arterioles involves both the Na^+^/K^+^-ATPase and inward-rectifier K^+^ channels. Microcirculation 2004; 11: 279–293. 15280082 10.1080/10739680490425985 PMC1382024

[28] Filosa JA Bonev AD Straub SV , et al. Local potassium signaling couples neuronal activity to vasodilation in the brain. Nat Neurosci 2006; 9: 1397–1403. 17013381 10.1038/nn1779

[29] Smith PD Brett SE Luykenaar KD , et al. K_IR_ channels function as electrical amplifiers in rat vascular smooth muscle. J Physiol 2008; 586: 1147–1160. 18063660 10.1113/jphysiol.2007.145474 PMC2375635

[30] Dawes M Sieniawska C Delves T , et al. Barium reduces resting blood flow and inhibits potassium-induced vasodilation in the human forearm. Circulation 2002; 105: 1323–1328. 11901043 10.1161/hc1102.105651

[31] Jantzi MC Brett SE Jackson WF Corteling R , et al. Inward rectifying potassium channels facilitate cell-to-cell communication in hamster retractor muscle feed arteries. Am J Physiol Heart Circ Physiol 2006; 291: H1319–H1328. 16617135 10.1152/ajpheart.00217.2006

[32] Liu GX Derst C Schlichthörl G , et al. Comparison of cloned Kir2 channels with native inward rectifier K^+^ channels from guinea‐pig cardiomyocytes. J Physiol 2001; 532: 115–126. 11283229 10.1111/j.1469-7793.2001.0115g.x PMC2278533

[33] Vanlandewijck M He L Mäe MA , et al. A molecular atlas of cell types and zonation in the brain vasculature. Nature 2018; 554: 475–480. 29443965 10.1038/nature25739

[34] He L Vanlandewijck M Mäe MA , et al. Single-cell RNA sequencing of mouse brain and lung vascular and vessel-associated cell types. Sci Data 2018; 5: 180160. 30129931 10.1038/sdata.2018.160 PMC6103262

[35] Bochaton-Piallat M-L Gabbiani F Ropraz P , et al. Cultured aortic smooth muscle cells from newborn and adult rats show distinct cytoskeletal features. Differentiation 1992; 49: 175–185. 1377654 10.1111/j.1432-0436.1992.tb00665.x

[36] Shanahan CM Weissberg PL. Smooth muscle cell heterogeneity. Arterioscler Thromb Vasc Biol 1998; 18: 333–338. 9514400 10.1161/01.atv.18.3.333

[37] Adam PJ Weissberg PL Cary NR , et al. Polyubiquitin is a new phenotypic marker of contractile vascular smooth muscle cells. Cardiovasc Res 1997; 33: 416–421. 9074707 10.1016/s0008-6363(96)00220-9

[38] Hoffman WE Edelman G Kochs E , et al. Cerebral autoregulation in awake versus isoflurane-anesthetized rats. Anesth Analg 1991; 73: 753–757. 1952176 10.1213/00000539-199112000-00013

[39] Wilde E Aubdool AA Thakore P , et al. Tail‐cuff technique and its influence on Central blood pressure in the mouse. Jaha 2017; 6. doi:10.1161/JAHA.116.005204. 10.1161/JAHA.116.005204 PMC5669161 28655735

[40] Leonoudakis D Conti LR Anderson S , et al. Protein trafficking and anchoring complexes revealed by proteomic analysis of inward rectifier potassium channel (Kir2.x)-associated proteins. J Biol Chem 2004; 279: 22331–22346. 15024025 10.1074/jbc.M400285200

