# Supplementary material for: Genetic ablation of smooth muscle KIR2.1 is inconsequential to the function of mouse cerebral arteries
Source: J Cereb Blood Flow Metab. 2022 Apr 11;42(9):1693–706. doi: 10.1177/0271678X221093432 (PMC9441723; doi:10.1177/0271678X221093432)
Supplement: sj-pdf-1-jcb-10.1177_0271678X221093432 - Supplemental material for Genetic ablation of smooth muscle KIR2.1 is inconsequential to the function of mouse cerebral arteries [file sj-pdf-1-jcb-10.1177_0271678X221093432.pdf]

Genetic ablation of smooth muscle $K_{IR}2.1$ is inconsequential to the function of mouse cerebral arteries

Paulina M. Kowalewska, Jacob Fletcher, William F. Jackson, Suzanne E. Brett, Michelle S.M. Kim, Galina Yu. Mironova, Nadia Haghbin, David M. Richter, Nathan R. Tykocki, Mark T. Nelson, Donald G. Welsh

Supplemental Methods

Superior epigastric artery smooth muscle electrophysiology

Superior epigastric arteries were enzymatically dissociated to yield isolated SMCs for perforated-patch recording of Ba^{2+}-sensitive K^+ currents to quantify the functional expression of SMC K_{IR} channels, and for qRTPCR as described.[1–3] After enzymatic isolation, a 100 μL aliquot of solution containing single SMCs was placed in the 1 mL recording chamber. The cells were allowed to settle and then were superfused with physiological salt solution (PSS in mM: 140 NaCl, 5 KCl, 1.8 $CaCl_2$, 1 $MgCl_2$, 10 4-(2-hydroxyethyl)-1-piperazineethanesulfonic acid (HEPES), 10 glucose; pH 7.4, 295 mOsm). Pipettes (2.5-4 MΩ when filled with pipette solution in mM: 100 K-aspartate, 43 KCl, 1 $MgCl_2$, 10 HEPES, 1 EGTA, 10 glucose, pH 7, containing 120-180 μg mL^{-1} Amphotericin B 120-180 μg mL^{-1}) were then applied to the surface of a cell with gentle suction to form a GΩ seal. After electrical access to a cell's cytoplasm was attained (< 20 MΩ access resistance usually within 30 min), cells were superfused with PSS containing 60 mM K^+ (to amplify K_{IR} currents, KCl substituted for NaCl in PSS). Cells were then held at -50 mV and subjected to 200 ms voltage ramps from -120 mV to +20 mV in the absence or presence of Ba^{2+} (100 μM) to assess functional expression of K_{IR} channels. All currents were normalized to cell capacitance which was determined using the membrane test utility in pClamp 10.2 via application of 10-mV depolarizing pulses from a holding potential of -50 mV and was 17.4 ± 2.6 pF (n = 6) for cells from C57BL/6 mice and 16.2 ± 1.6 pF (n = 10) for cells from SMC $K_{IR}2.1^{-/-}$ (P = 0.7144; unpaired *t*-test for unequal variance).

References

1 Hayoz S, Bradley V, Boerman EM, Nourian Z, Segal SS, Jackson WF. Aging increases capacitance and spontaneous transient outward current amplitude of smooth muscle cells from murine superior epigastric arteries. *Am J Physiol Circ Physiol* 2014; **306**: H1512–H1524.

2 Hayoz S, Pettis J, Bradley V, Segal SS, Jackson WF. Increased amplitude of inward rectifier K^+ currents with advanced age in smooth muscle cells of murine superior epigastric arteries. *Am J Physiol Circ Physiol* 2017; **312**: H1203–H1214.

3 Mullan B, Pettis J, Jackson WF. T-type voltage-gated Ca^{2+} channels do not contribute to the negative feedback regulation of myogenic tone in murine superior epigastric arteries. *Pharmacol Res Perspect* 2017; **5**: e00320.

Supplemental Figures

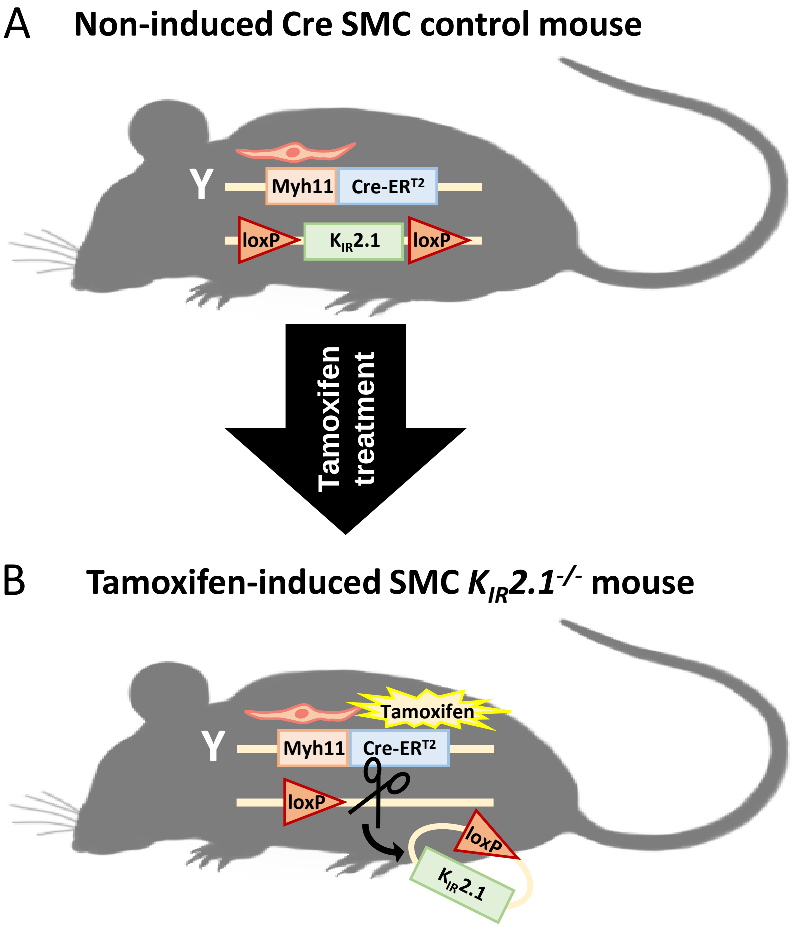

Supplemental Figure S1. Generation of smooth muscle cell specific K_{IR}2.1 knockout mice. (A) Control mice, designated as "non-induced Cre SMC controls", possess a Cre recombinase gene under the control of myosin, heavy polypeptide 11, smooth muscle (*Myh11*) promoter/enhancer inserted on the Y chromosome. *Myh11* encodes a contractile protein expressed exclusively in smooth muscle cells. The Cre recombinase is fused to a human estrogen receptor mutant that binds the synthetic ligand tamoxifen but does not bind its natural ligand 17β-estradiol at physiological concentrations. The K_{IR}2.1 gene in these mice is flanked by *loxP* sequences. (B) Treatment with tamoxifen allows the fused Cre recombinase/estrogen receptor to move from cytoplasm to the nuclear compartment. The tamoxifen-induced, Cre-mediated recombination results in deletion of the floxed K_{IR}2.1 gene in smooth muscle cells. These mice are designated as "tamoxifen-induced SMC $K_{IR}2.1^{-/-}$ mice."

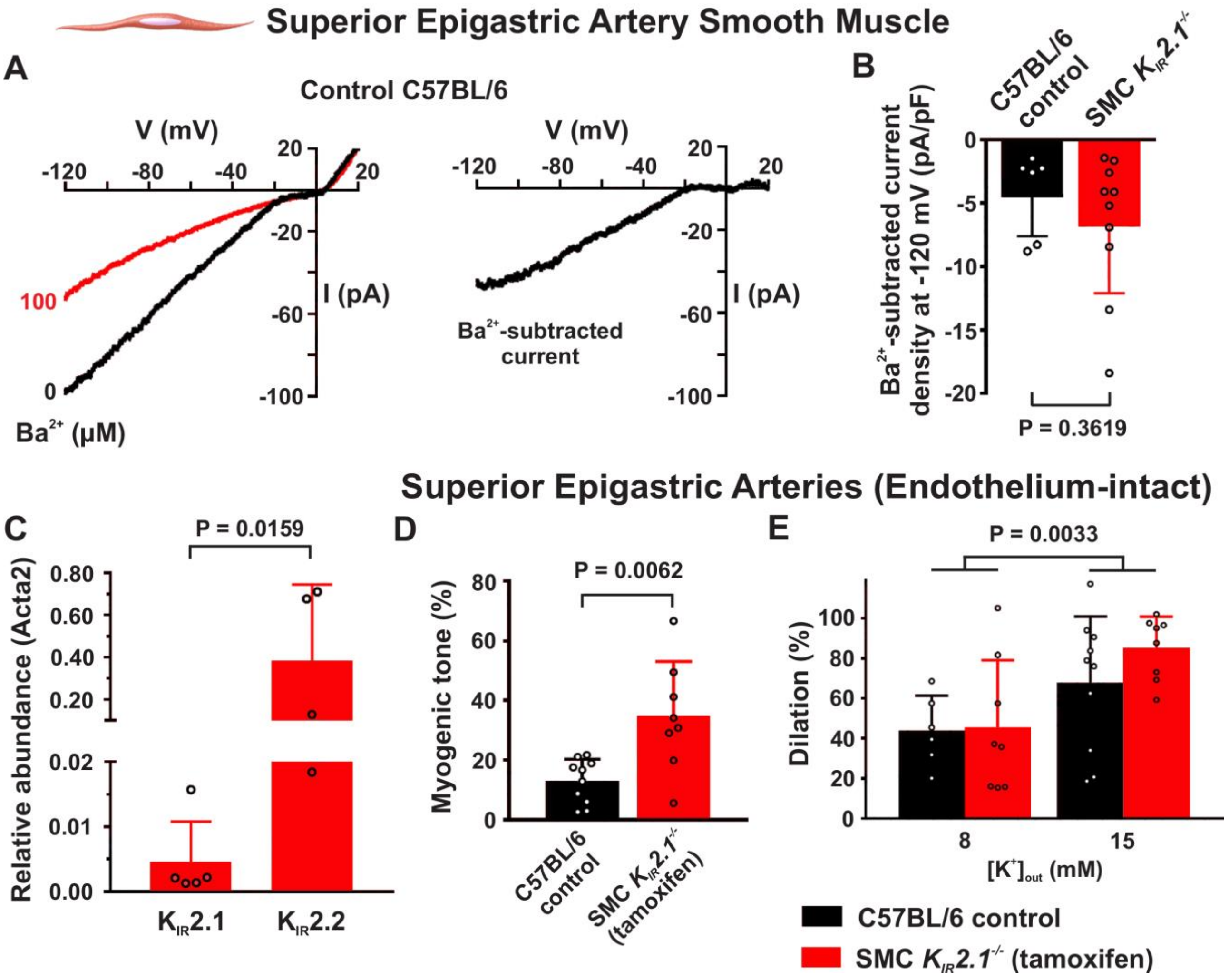

Supplemental Figure S2. Smooth muscle knockout of K_{IR}2.1 does not eliminate K_{IR} currents in superior epigastric artery (SEA) smooth muscle cells (SMCs). (A) Typical perforated-patch, whole-cell recording of K_{IR} currents from a SEA SMC in the absence and presence of 100 μM Ba^{2+} is shown with Ba^{2+}-subtracted current. (B) Summary data for Ba^{2+}-subtracted current density recorded at -120 mV for SEA SMCs from C57BL/6 (n = 6 cells from 3 mice) and SMC $K_{IR}2.1^{-/-}$ mice (n = 10 cells from 4 mice). Ba^{2+}-sensitive currents were similar in SMCs from C57BL/6 and SMC $K_{IR}2.1^{-/-}$ mice (unpaired t-test). (C) K_{IR}2.2 is prominently expressed in SEA SMCs. Graph shows mRNA expression relative to α-smooth muscle actin (Acta2) in $K_{IR}2.1^{-/-}$ SMCs (n = 5 isolates for K_{IR}2.1 and n = 4 isolates for K_{IR}2.2; Mann-Whitney U-test). (D) Myogenic tone in tamoxifen-induced SMC $K_{IR}2.1^{-/-}$ arteries (n = 8 vessels) was significantly greater than that observed in vessels from C57BL/6 (n = 10 vessels; Mann-Whitney U-test). (E) Dilation was induced by increasing $[K^+]_{out}$ from 5 mM to 8 mM or 15 mM. Two-way ANOVA indicated significant K^+-induced dilation (P = 0.0033), but no significant differences in response between mouse strains (P = 0.3393). In the presence of 100 μM Ba^{2+}, K^+-induced dilation of arteries from SMC $K_{IR}2.1^{-/-}$ was reduced to 4.3 ± 4.5 % dilation in 8 mM K^+ (P = 0.0007 vs. control, n = 8) and 7.2 ± 2.5 % dilation in 15 mM K^+ (P < 0.0001 vs. control, n = 8 vessels) establishing the Ba^{2+} sensitivity of these responses as previously reported in SEAs from C57BL/6 mice [23]. K^+-induced dilation was computed as (peak K^+-induced diameter – resting diameter)/(0-Ca^{2+} diameter – resting diameter).
