# Supplementary material for: Genetic ablation of smooth muscle KIR2.1 is inconsequential to the function of mouse cerebral arteries
Source: J Cereb Blood Flow Metab. 2022 Apr 11;42(9):1693–706. doi: 10.1177/0271678X221093432 (PMC9441723; doi:10.1177/0271678X221093432)

Genetic ablation of smooth muscle K_{IR}2.1 is inconsequential to the function of mouse cerebral arteries

Paulina M. Kowalewska, Jacob Fletcher, William F. Jackson, Suzanne E. Brett, Michelle S.M. Kim, Galina Yu. Mironova, Nadia Haghbin, David M. Richter, Nathan R. Tykocki, Mark T. Nelson, Donald G. Welsh

Supplemental material: full unedited blots for figure 3

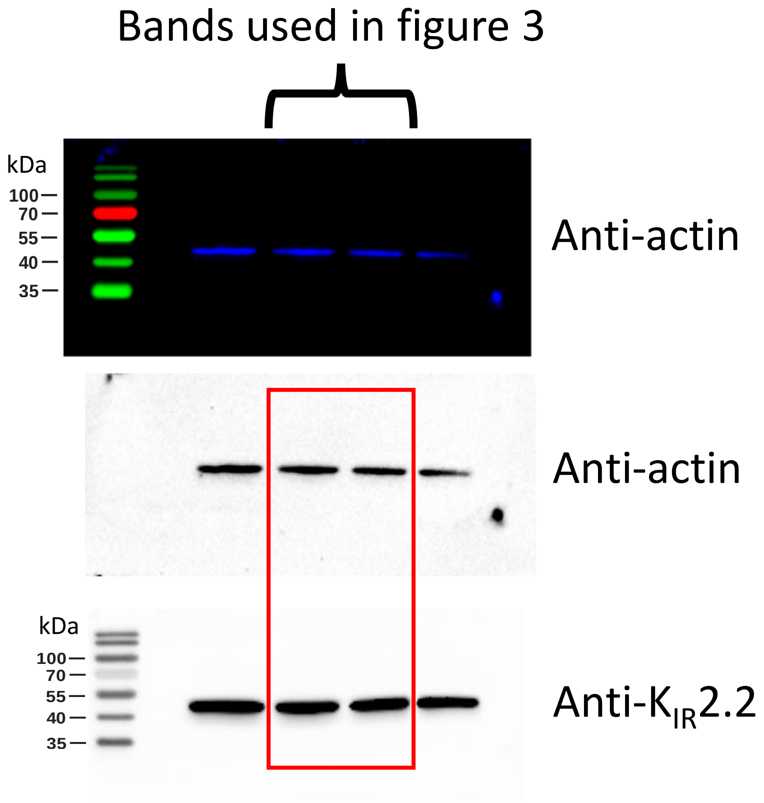

Supplement: sj-pdf-2-jcb-10.1177_0271678X221093432 - Supplemental material for Genetic ablation of smooth muscle KIR2.1 is inconsequential to the function of mouse cerebral arteries [file sj-pdf-2-jcb-10.1177_0271678X221093432.pdf]
